# Hypertonic saline infusion suppresses apoptosis of hippocampal cells in a rat model of cardiopulmonary resuscitation

**DOI:** 10.1038/s41598-017-05919-4

**Published:** 2017-07-19

**Authors:** Xiang Zhou, Yong Liu, Yang Huang, ShuiBo Zhu, Jian Zhu, RongPing Wang

**Affiliations:** 1 Southern Medical University, Guangzhou, China; 2 Department of Thoracic Cardiovascular Surgery, Wuhan General Hospital of People’s Liberation Army of China, Guangzhou, China

## Abstract

Hypertonic saline (HS) attenuates cerebral edema, improves microcirculation perfusion and alleviates inflammation. However, whether the beneficial effect of HS on neurological function after cardiopulmonary resuscitation (CPR) in rat model of asphyxial cardiac arrest (CA) is mediated via attenuating apoptosis of neurons is not known. We studied the neuroprotective effect of HS in rats after CA and CPR, and explored the likely underlying mechanisms. Animals were randomly assigned to 4 equal groups (n = 15 each) according to the different infusions administered during resuscitation: control (C), normal saline (NS), hypertonic saline (HS), and hydroxyethyl starch (HES) groups. NDS at 12, 24, 48 and 72 h post-ROSC in the HS group were significantly higher than those in the NS and HES groups. Western blot analysis demonstrated a significant increase in Bcl-2 expression in HS, as compared to that in the NS and HES groups. However, Bax and Caspase-3 expressions in HS were significantly lower than that in the NS and HES groups. The apoptosis rate in HS was significantly lower than that in the NS and HES groups, suggesting HS treatment during resuscitation could effectively suppress neuronal cell apoptosis in hippocampal CA1 post-ROSC and improve neuronal function.

## Introduction

An estimated 54.4 million people sustain cardiac arrest (CA) in China each year^1^, of which only about 1% people survive^2^. According to a large multicenter registry-based observational study, up to 27% of all survivors of CPR sustain some form of neurological deficit^3^. The pathophysiologic mechanisms of brain injury following CA are complex and multifactorial, including the primary ischemic neuronal injury and secondary injury from consequent cerebral edema^4^. Therefore, it is very important to protect brain function during the advanced life support (ALS) treatment after CA and CPR. Many studies have indicated that neuronal apoptosis is one of underlying mechanisms of post-CA and post-CPR neuronal cell death^5, 6^. Ischemia during cardiac arrest and after restoration of spontaneous circulation (ROSC) will lead to neuronal necrosis, also reperfusion will aggravate this ischemic injury. Therefore, attenuation of neuronal apoptosis is a key strategy to maintain neurological function in these patients^7^.

Hypertonic saline is known to reduce intracranial pressure, promote osmotic diuresis and improve brain perfusion^8^. In addition, it can regulate immune function and has anti-inflammatory effect^9^. Both experimental (animal studies)^10^ and clinical research^11^ has shown hypertonic saline to increase the survival rates after CA and CPR and to improve neuronal function^12^. However, the underlying mechanism of this neuroprotective effect is not completely clear. Majority of research conducted till date has centered on the effect of hypertonic saline on inflammatory biomarkers and cerebral stroke area in focal brain ischemic or shock model^9, 13, 14^. However, the patho-mechanism of global cerebral ischemic insults resulting from asphyxia of CA is inherently different from focal cerebral impairment caused by brain artery occlusion^15^. Protecting brain cells by therapeutic methods such as mild hypothermia have been shown to suppress apoptosis^5^ and ameliorate neuronal damage^16^. Rüdiger *et al*. reported that hypertonic saline hydroxyethyl starch failed to ameliorate the newly generated hippocampal neurons in the survived CA and CPR rat models^17^. To date, it is not known whether hypertonic saline improves neurological function by attenuating apoptosis of brain injury after resuscitation in an asphyxial cardiac arrest rat model.

In the present study, we investigated the neuroprotective effect of 10% hypertonic saline on the rats after CA and CPR, and also attempted to explore the likely underlying mechanism.

## Materials and Methods

### Animal Preparation

After approval of the animal ethics committee and the animal care committee at the Wuhan general hospital of Chinese PLA (WH2015-13), 60 male Sprague-Dawley rats (250–300 g, Certificate No. 42009800001607) were provided by the Experimental Animal Center of Tongji Medical College, Huazhong University of Science and Technology. All rats were housed in a controlled environment (temperature 22 °C; free access to water and food and 12 hours’ light and 12 hours’ dark cycle. All rat treatment and care in details were in agreement with international institutional guidelines^18^.

### Asphyxial cardiac arrest model

Experimental protocols on rats were performed based on Utstein-style guidelines for Uniform Reporting of Laboratory CPR Research^19^ and partly modified. In brief, each rat was anesthetized using 10% chloral hydrate (0.3 mL/100 g) intraperitoneal injection. A rectal probe was inserted and rectal temperature of rats was maintained at about 36 ± 1 °C during surgery with a biofeedback-heating mat under operating platform. The right femoral artery and vein were exposed via a skin incision along the right groin and blunt dissection of the subcutaneous tissue. Venous indwelling catheter (24G) was placed in the femoral artery and connected to a pressure transducer. Powerlab 16/30 (AD-Instruments, Australia) was used to monitor arterial blood pressure and electrocardiogram of normal lead II continuously. A 24G venous indwelling catheter was also inserted in the right femoral vein for continuous administration of fluid infusion via micro-perfusion pump at a rate of 2 mL/h. After orotracheal intubation using a teleflex of 16G venous indwelling catheter, median incision of neck was made on each rat, and blunt dissection of subcutaneous tissue performed to expose the trachea. In order to prevent air leakage, a firm ligature was placed on the trachea during mechanical ventilation of the rats (respiratory frequency 70 bpm, tidal volume 4 mL/100 g) by ventilator (ALC-V, Shanghai Alcott Biological Technology Co., Ltd.).

Under deep anesthesia, rats were prepared for the next experimental procedure. After 30 mins of venous infusion of different kinds of fluids (0.9% normal saline for C and NS groups, 10% hypertonic saline and hydroxyethyl starch 130/0.4 for HS and HES groups, respectively) and 10 minutes of mechanical ventilation, asphyxia was induced in rats by stopping the ventilator. At the same time, the tracheal tubes were also clamped tightly. CA was defined as the systolic blood pressure (SBP) < 25 mm Hg^19^. CPR was initiated after 5 mins of CA. Controlled ventilation was initiated at the same respiratory frequency of 70 bpm and tidal volume 4 mL/100 g, as administered earlier. External chest compressions were carried out with the instruction rhythm of metronome at the frequency of 200 compressions/min and the compression depth was 1/3 diameter of rat thorax anterior to posterior.

Adrenaline was injected (4 μg/100 g) through the right femoral venous indwelling catheter after 10 seconds of manual thoracic compressions. Restoration of spontaneous circulation (ROSC) was defined as the return of spontaneous sinus rhythm, with SBP > 60 mmHg which was maintained for at least 10 minutes. If ROSC was not achieved, animals were declared dead and excluded from the next experimental protocol. Controlled ventilation was continued for rats who survived the CA and CPR. Spontaneous respiration was carefully monitored every 5 minutes. If spontaneous respiration was good enough to be weaned from ventilator, the orotracheal tube was removed and the neck incision was closed. Venous indwelling catheters were withdrawn from the right femoral artery and vein, and venous infusion was stopped.

## Experimental

### Groups

Animals were divided into 4 groups (n = 15 for each group) using computer generated random numbers: C group (control group), NS group (normal saline group), HS group (hypertonic saline group) and HES group (hydroxyethyl starch group). Animals in the control group underwent anesthesia, venous infusion (2 mL/h) of normal saline and sham-operation, including placement of indwelling catheters in femoral artery and vein, orotracheal intubation and neck incision for blunt dissection of subcutaneous tissue which was similar to the experimental procedures, except CA and CPR procedures and adrenaline application. After surgery, all rats were subcutaneously injected with 1 mL of 5% Glucose Solution (GS) in the abdominal wall and placed in a 23 °C incubator with free access to food and water. Based on randomization, 30 mins before CA, rats in the NS, HS and HES groups received different venous infusions (2 mL/h) (normal saline (0.9%), hypertonic saline (10%) and hydroxyethyl starch (6%), respectively).

### Basic measurements

Introperative monitoring of blood pressure, ECG, heart rate and rectal temperature was performed using Powerlab. The survival rates of CPR, as well as that at 6 h, 24 h, 48 h and 72 h after surgery were recorded.

### Evaluation of neurologic deficit

Neurological examination and evaluation of neurological deficit was performed using neurological deficit scores (NDS) by an investigator who was blinded to the experimental design. NDS of the survived rats was assessed during the recovery period at 6, 12, 24, 48 and 72 hours after ROSC. NDS were determined on a scale of 0–80, on the basis of arousal level, cranial nerve reflexes, muscle tone, motor function, seizure and simple behavioral responses^17, 20^. Score 80 represented normal performances (no deficit); score 0 represented the brain death (most severe neurological deficit).

### Hematoxylin and eosin (H&E) staining for hippocampus CA1 area

Euthanasia was performed 72 hrs after surgery under deep anesthesia. A whole brain was removed; hippocampus was isolated carefully and placed in 4% paraformaldehyde solution for further fixation for 24 hours. After conventional gradient ethanol dehydration, brain tissues was rendered transparent by dimethylbenzene and embedded in paraffin. Parallel coronal sections (4-μm thick) of paraffin-embedded brain tissue were prepared for immunohistochemical examination. Tissue sections were immersed in hematoxylin solution for 5–7 mins and washed in running water. Section differentiation was performed with acid alcohol (0.3%) and washed in running water again to remove excess dye. Sections were then stained with eosin solution (1%) for 2 mins and rinsed with running water for 30 seconds, repeatedly. Finally, brain tissue sections stained with H&E were sealed up with neutral balsam for visualization on biomicroscopy (NIKON, E100, Japan), after anhydrous alcohol dehydration, vitrification by dimethylbenzene and drying.

### Western blot analysis for expression of apoptosis protein

Total protein was extracted from hippocampal tissue samples of euthanized rats 72 hrs after surgery, which were cryopreserved in liquid nitrogen (−196 °C). BCA Protein Assay Kit (Pierce, USA) was used to detect protein concentrations. Equal amounts of protein (25 μg) were separated by sodium dodecyl sulfate polyacrylamide gel electrophoresis (SDS–PAGE) and transferred to Polyvinylidene difluoride (PVDF) membrane (Millipore, USA), which was blocked with 5% nonfat milk for 2 hr. Then membranes were incubated with primary antibodies Bax, Bcl-2 and Caspase-3 (Proteintech, Wuhan, China. 1:1000) overnight at 4 °C. After washing with 1 × Tris-Buffered Saline and Tween 20 (TBST) 3 times, membranes were incubated with horseradish peroxidase-conjugated secondary antibodies (Boster, Wuhan, China) at 37 °C for 2 hrs, then incubated shakily with horseradish peroxidase-conjugated goat anti-rabbit secondary antibody (1:50000, Abcam, Britain) for 2 hrs. Visualization was performed using an enhanced chemiluminescence detetion kit (Pierce, USA), and relative band intensities were quantified by use of BandScan (Glyko, ProZyme, USA).

### Determination of apoptosis of hippocampal neurons in brain tissue by Tunel assay

Apoptosis of hippocampal neurons after resuscitation was detected by terminal deoxynucleotidyl transferase-mediated uridine 59-triphosphate-biotin nick end labeling (Tunel) assay. Coronal paraffin wax brain tissue sections were obtained as previously described^21^. In order to measure the apoptosis of hippocampal neurons, *In Situ* Cell Death Detection Kit (Roche, Switzerland) was used according to the manufacturer’s instructions. Apoptotic changes were detected via fluorescence microscopy (Olympus, Tokyo, Japan). Positive signal was indicated by green fluorescence staining region which represented apoptotic cells and blue fluorescence was stained by DAPI which represented cell nuclei. The images were obtained using a light microscope (Olympus, Japan). The apoptotic rate was defined as the ratio of green staining cells to blue staining cells. Three regions of interest were randomly chosen in each hippocampal CA1 section, the apoptotic rate calculated according to the formula mentioned above; mean of three measurements of apoptotic rate are presented.

### Flow Cytometric Apoptosis Assays

72 hrs after CA and CPR, hippocampal tissue of rat was taken for preparation of flow cytometry apoptosis assays. Cells were collected after trypsinization. The apoptosis assays were measured by flow cytometers (FCM, BD Biosciences, USA) with Annexin V-FITC Apoptosis Detection Kit (KeyGEN BioTECH, Nanjing, China) according to the instruction of manufacturer.

### Statistical analysis

A database was built using SPSS 20.0 for statistical analysis. Descriptive data are presented as mean ± SD or median (interquartile range) for continuous variables; categorical variables are presented as frequencies. Descriptive data were analyzed by repeated measures Analysis of Variance (ANOVA) or one-way ANOVA; Fisher exact test (Two-tailed) was used for categorical variables. *P* < 0.05 was considered as statistically significant.

## Results

### Resuscitation success and survival rate

There was no significant difference in resuscitation success rate between NS, HS and HES groups (*P* > 0.05, respectively). All 15 rats in the control group survived 72 hrs after surgery. Survival rates at 24, 48 and 72 hours after surgery in the NS group were significantly lower than those in the control group (*P* < 0.05). Survival rates at 48 and 72 hrs after surgery in the HES group were significantly lower than those in the control group (both *P* < 0.05). In contrast, no significant difference in survival rates at 6, 24, 48 and 72 hours was observed between the HS and control groups (*P* > 0.05, respectively; Table 1).

**Table 1:** Success rate for CPR and survival rates post-surgery in the study rats.

	CPR success rate (n %)	6 hrs survival rate (n %)	24 hrs survival rate (n %)	48 hrs survival rate (n %)	72 hrs survival rate (n %)
C group	15 (100.00%)	15/100.00%	15/100.00%	15/100.00%	15/100.00%
NS group	12 (80.00%)	11/73.33%	10/66.67%^★^	10/66.67%^★^	10/66.67%^★^
HS group	13 (87.00%)	13/86.67%	12/80.00%	12/80.00%	12/80.00%
HES group	12 (80.00%)	12/80.00%	11/73.33%	10/66.67%^★^	10/66.67%^★^

### Rat NDS scores post-ROSC in 4 groups

**Figure 1:**
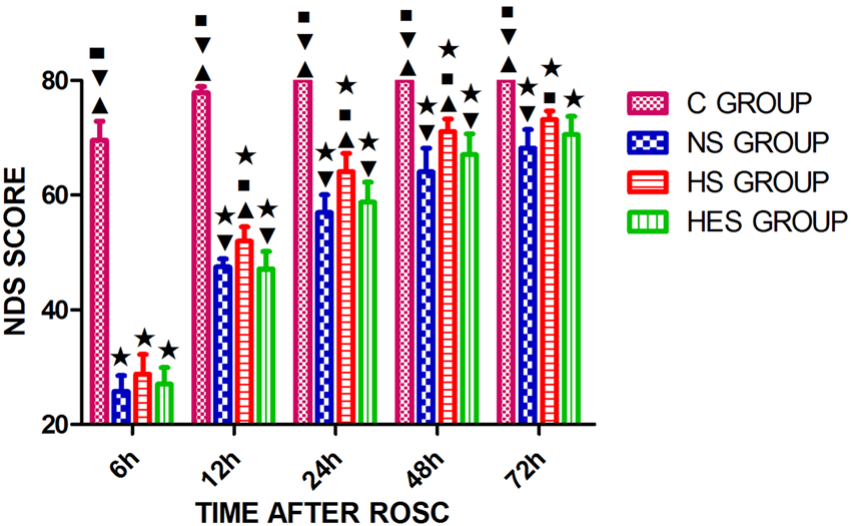
Survival rate and neurological deficit evaluation at various time-points during cardiopulmonary resuscitation (CPR) and after ROSC. NDS of the survived rats assessed during the recovery period at 6, 12, 24, 48 and 72 hours after ROSC. NDS were determined by a scale of 0–80, based on arousal levels: cranial nerve reflexes, muscle tone, motor function, seizure and simple behavioral responses. Score 80 represented normal performances (no deficit); score 0 represented the brain death (most severe neurological deficit). ^★^ *P* < 0.05 vs control group; ^◾^ *P* < 0.05 vs NS group; ^▾^ *P* < 0.05 vs HS group; ^▴^ *P* < 0.05 vs HES group.

NDS at 6, 12, 24, 48, and 72 hours post-ROSC in the NS, HS and HES groups were significantly lower than that in the C group (*P* < 0.001, respectively); NDS at 12, 24 and 48 hours in the HS group were significantly higher than those in the NS and HES groups (12 h: *P*1 = 0.001, *P*2 = 0.007; 24 h: *P* < 0.001, *P*2 = 0.012; 48 h: *P*1 = 0.002; *P*2 = 0.046); NDS at 72 hours in the HS group were significantly higher than those in the NS group (*P* = 0.004. Fig. 1).

### Changes in mean arterial pressure (MAP) at different time points

**Figure 2:**
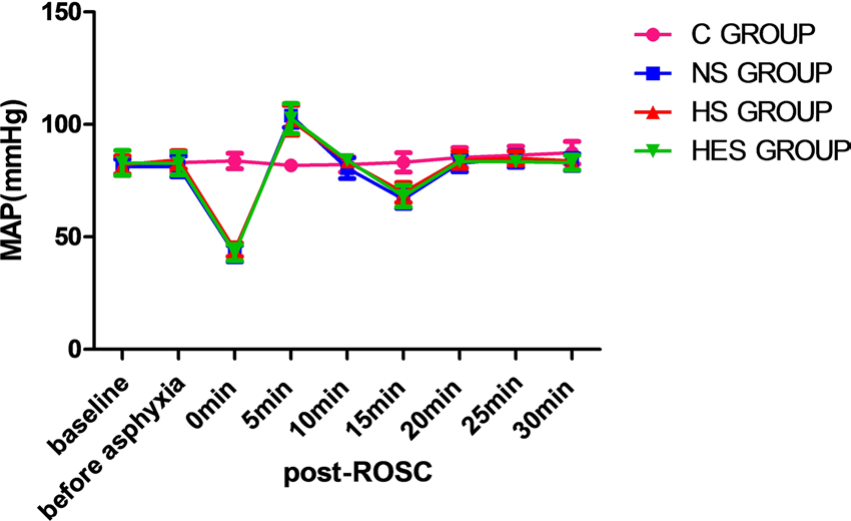
Changes of mean arterial pressure (MAP) in C, NS, HS and HES groups during cardiopulmonary resuscitation (CPR) and after ROSC. MAP of different groups at baseline, before asphyxia, and different time points after ROSC. 0 min after ROSC means the time that return of spontaneous sinus rhythm. All the MAP were measured by Powerlab 16/30 from femoral artery.

No significant between-group difference with respect to MAP was observed at baseline, before asphyxia, and at 20 and 25 minutes post-ROSC between the C, NS, HS and HES groups (*P* > 0.05); however, the immediate MAP at 0 min and 15 min post-ROSC in the NS, HS and HES groups, were significantly lower than that in the control group (*P* < 0.001, respectively). MAP at 5 min post-ROSC in the NS, HS and HES groups were significantly higher than that in the control group (P < 0.001, respectively). MAP at 10 min post-ROSC in the HS and HES groups were higher than that in the NS group (*P* < 0.05, respectively). Similarly, MAP at 30 min post-ROSC in the NS, HS and HES groups was significantly lower than that in the control group (*P* < 0.05, respectively. Fig. 2).

### Duration of different phases and volume of fluid infused in each phase of the establishment of rat model of CPR

There were no significant differences with respect to duration of the key phases of CPR, including time for establishment of CA model; duration of asphyxia and CPR; duration of mechanical ventilation and total time between the NS, HS and HES groups (*P* > 0.05, respectively). Further, there was no significant different between these 3 groups with respect to the volume of fluid injected by microinfusion pump during the experiment (*P* > 0.05 for all; Table 2).

**Table 2:** Duration of different phases and volume of fluid infused in each phase of the establishment of rat model of CPR.

Study groups	Time duration for establishment of CA model (min)	Duration of asphyxia (min)	Duration of CPR (min)	Duration of mechanical ventilation (min)	Total time (min)	Infusion volume (mL)
NS	31.11 ± 0.86	11.62 ± 0.63	1.09 ± 0.25	44.37 ± 3.94	101.40 ± 6.97	1.82 ± 0.14
HS	30.76 ± 2.11	11.18 ± 0.97	0.98 ± 0.23	44.02 ± 4.42	102.34 ± 5.29	1.83 ± 0.13
HES	31.54 ± 2.62	10.93 ± 0.91	1.06 ± 0.25	43.63 ± 4.28	102.09 ± 5.75	1.85 ± 0.12

*P* > 0.05 for all. Time duration for establishment of CA model, from beginning of anesthesia to completion of blunt dissection of trachea in the neck median incision; Duration of asphyxia, from clamping the tracheal tube to cardiac arrest; Duration of CPR, from performing external chest compressions to restoration of spontaneous circulation (ROSC); Total time, from beginning of anesthesia to removal the orotracheal tube and closing the neck incision; Infusion volume, the liquid volume injected by microinfusion pump during the whole experimental procedure.

### Pathological changes of Hippocampal CA1 by H&E staining

**Figure 3:**
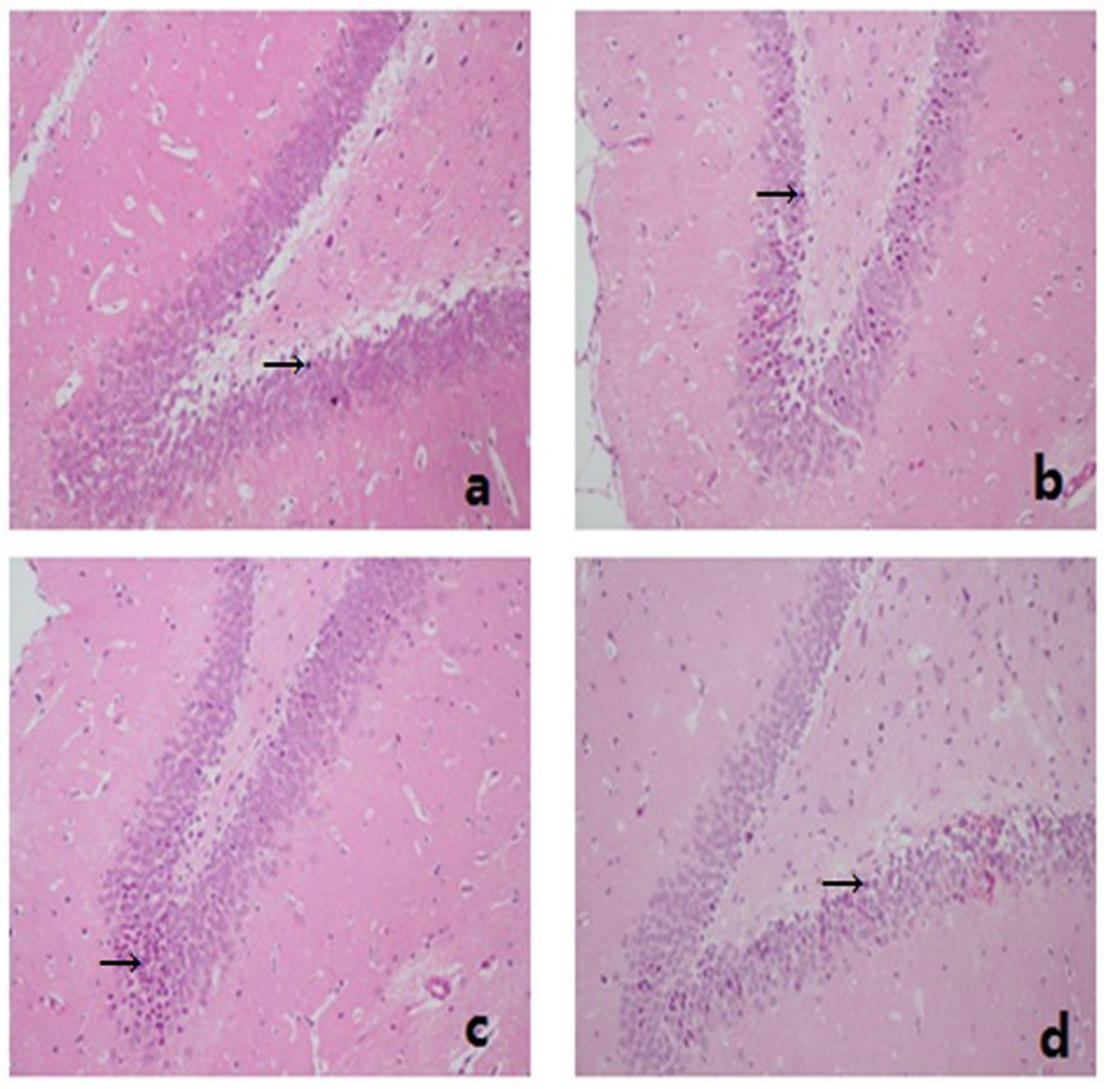
Histopathological examination of H&E stained sections of Hippocampal CA1. Neuronal cells in Hippocampal CA1 in control group (**a**); NS (**b**); HS (**c**) and HES groups (**d**). Black arrow points neuronal cells with nuclear staining was purple red with chromatin condensation. magnification, 200x.

Neuronal cells in Hippocampal CA1 in control group were well-formed, round or oval in shape and -arranged well. Nuclear staining was weak and the chromatin was relatively dispersed. (Fig. 3a). However, the neuronal cells in the NS and HES groups were arranged in significantly disordered manner. Nuclear staining was purple red with chromatin condensation. Obvious cell swelling, degeneration and necrosis of cells were observed (Fig. 3b and d). Contrarily, cells in the HS group were arranged with a little disorder. Nuclear staining was purple red with chromatin condensation with a moderate number of degenerative cells. Mild to moderate degree of cell swelling was observed (Fig. 3c).

### Effect on apoptosis protein expression of Bcl-2, Bax and Caspase-3

**Figure 4:**
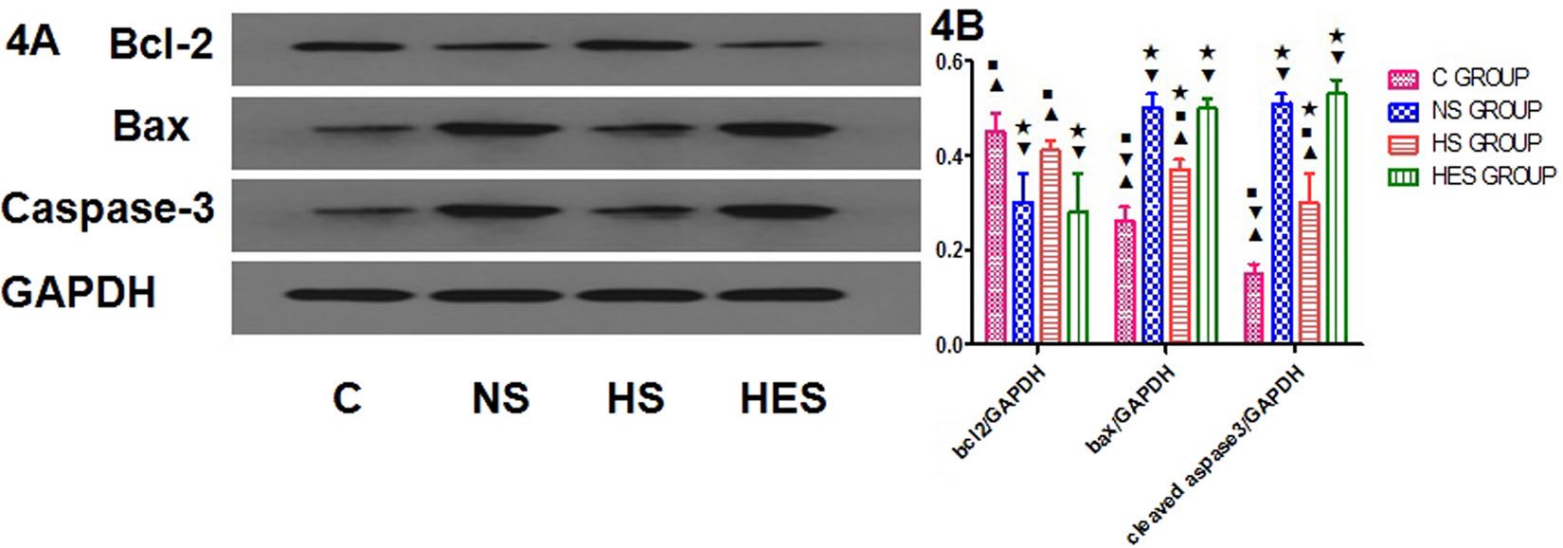
Expression of apoptosis protein, Bcl-2, Bax and Caspase-3 by Western bolt. Apoptosis protein Bcl-2, Bax and Caspase-3 by Western blot analysis (Fig. 4A, cropped gels/blots, full-length gels/blots are included in the Supplementary Information file) and quantitative analysis (Fig. 4B). ^★^ *P* < 0.05 vs control group; ^◾^ *P* < 0.05 vs NS group; ^▾^ *P* < 0.05 vs HS group; ^▴^ *P* < 0.05 vs HES group.

Different infusion fluids used in the experiment appeared to have different effects on the expression of apoptosis protein, Bcl-2, Bax and Caspase-3. Western blot analysis demonstrated a significant decrease in Bcl-2 expression in the NS and HES groups (*P* < 0.001 vs. control for both groups). In contrast, a significant increase in Bcl-2 expression was observed in HS group as compared to that in the NS and HES groups (both *P* < 0.01), while there was no difference with that in the control group (*P* = 0.432). Bax expression levels in NS, HS and HES were significant higher than that in the control group (*P* < 0.001 for all three groups). Bax expression in the HS group was significantly lower than that in the NS and HES groups (*P* < 0.001 for both). Caspase-3 expression levels in NS, HS and HES were significantly higher than that in the control group (*P* < 0.001, for all 3 groups). However, Caspase-3 expression in HS was significant lower than that in the HS and HES groups, (*P* < 0.001 for both respectively, Fig. 4A and B).

### Evaluation of CA-induced neuronal cell loss

#### TUNEL assay of Hippocampal CA1 neuronal cells

**Figure 5:**
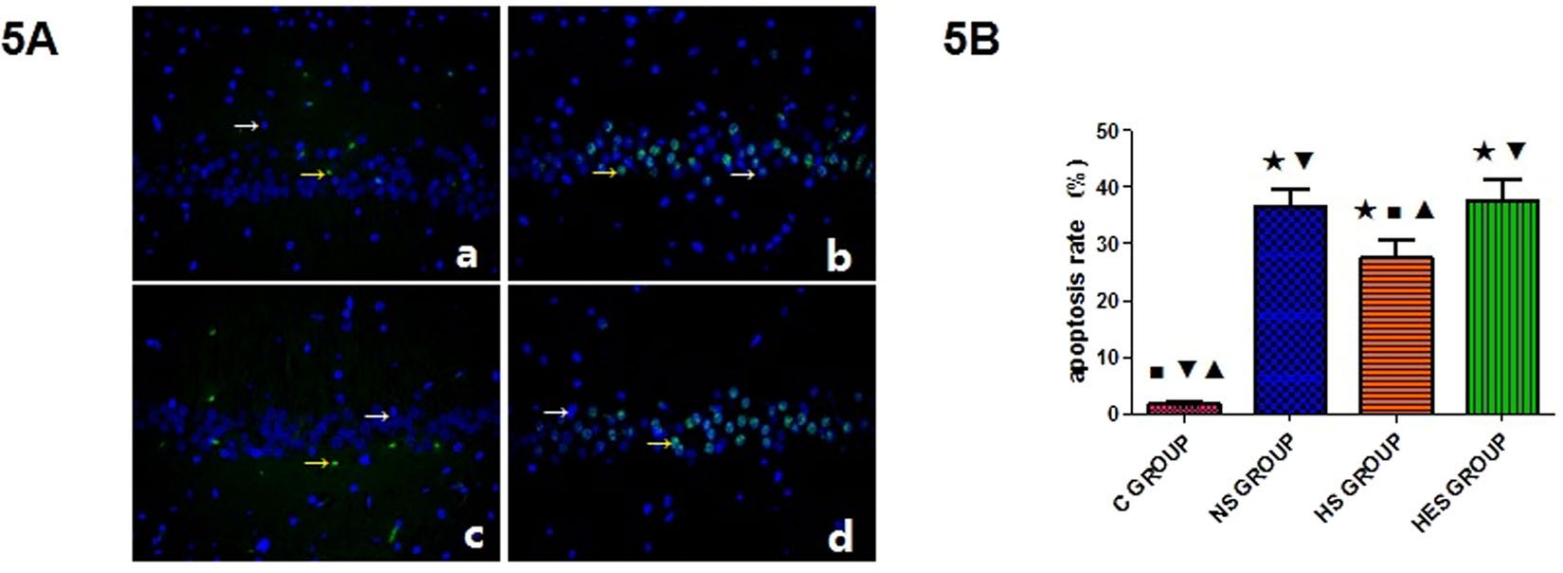
Hippocampal CA1 neuronal cell apoptotic rate by TUNEL. (**A**) Immunofluorescence staining for assessment of neuronal cell apoptosis in hippocampal CA1 at 72 hrs after ROSC in control (a), NS (b), HS (c) and HES (d) groups; Green stained cells are apoptotic cells and blue-stained region was cell nuclei; magnification, 400x. (**B**) Results of quantitative analysis for calculation of neuronal cell apoptosis rate. Yellow arrow points green fluorescence staining region which represented apoptotic cells and white arrow points blue fluorescence which represented cell nuclei. ^★^ *P* < 0.05 vs control group; ^◾^ *P* < 0.05 vs NS group; ^▾^ *P* < 0.05 vs HS group; ^▴^ *P* < 0.05 vs HES group.

At 72 hrs after ROSC, apoptotic rate of neuronal cells in hippocampal CA1 in the NS, HS and HES groups were significantly higher than that in the control (*P* < 0.001 for all). However, the apoptotic rate in HS was significantly lower than that in the NS and HES groups (*P* < 0.001 for both; Fig. 5A and B).

#### Hippocampal CA1 neuronal cell apoptotic rate by Flow cytometry assay

**Figure 6:**
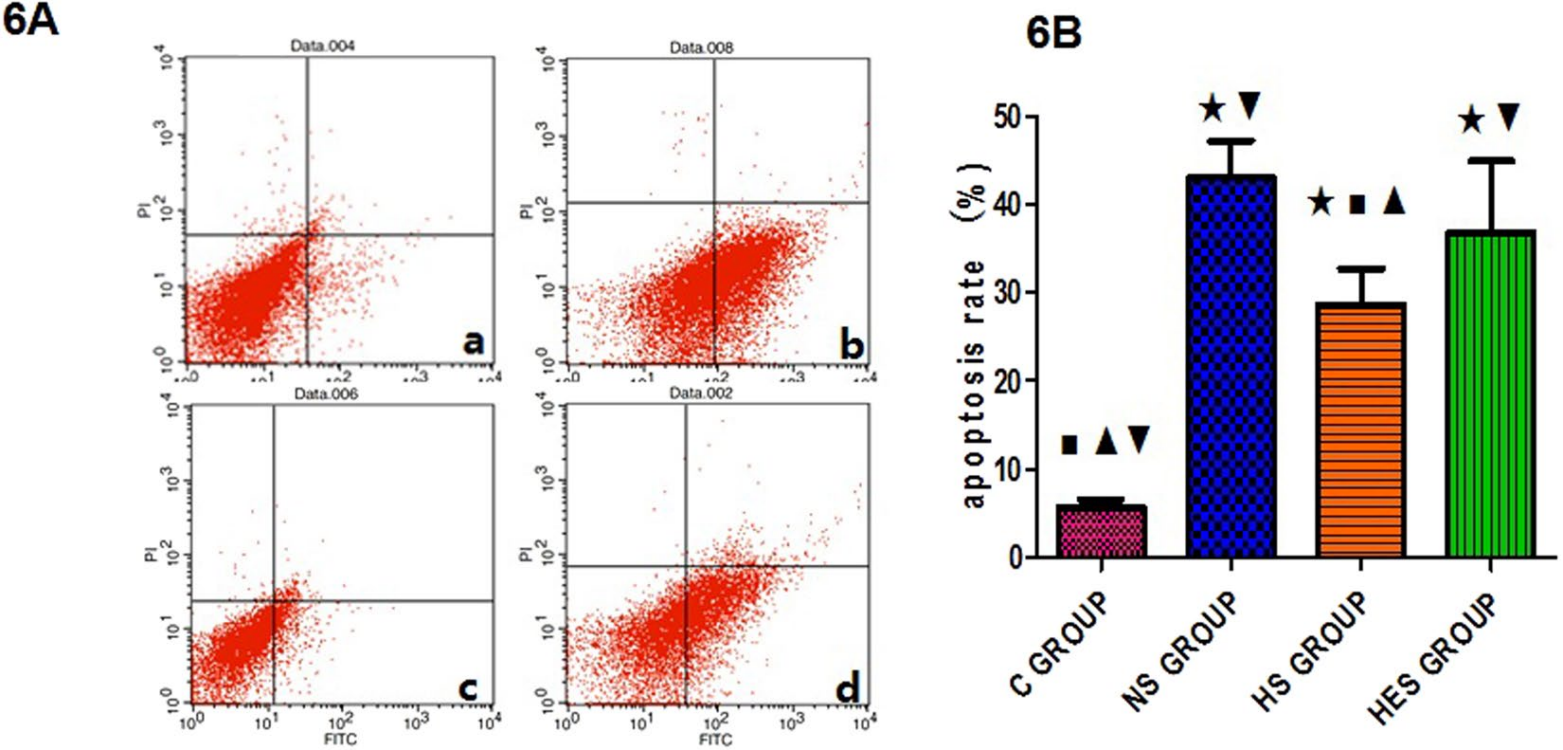
Hippocampal CA1 neuronal apoptosis cellS by Flow cytometry assay. (**A**) Hippocampal CA1 neuronal apoptosis cells by Flow cytometry assay in NS, HS, HES and control (C) groups. Lower left quadrant means normal cell, upper left quadrant means necrotic cell, lower right quadrant means early apoptotic cell and upper right quadrant means late apoptotic cell. Apoptosis cells include both early and late apoptotic cels. (**B**) Quantitative analysis results of neuronal cell apoptosis rate. ^★^ *P* < 0.05 vs control group; ^◼^ *P* < 0.05 vs NS group; ^▾^ *P* < 0.05 vs HS group; ^▴^ *P* < 0.05 vs HES group.

The apoptosis rates of neuronal cells in hippocampal CA1, as assessed on flow cytometry assay, in the NS, HS and HES groups were significantly higher than that in the control group (*P* < 0.001 for all). However, the apoptotic rate in HS group was lower than that in the NS and HES groups (*P* < 0.001 and *P* = 0.047, respectively; Fig. 6A and B).

## Discussion

In the present study, use of hypertonic saline infusion during resuscitation significantly inhibited the expression of apoptosis proteins Bax, and Caspase-3 and enhanced the expression of anti- apoptosis protein Bcl-2. Further, hypertonic saline infusion significantly decreased the apoptotic rate of CA-induced hippocampal neuronal cells 72 hrs post-ROSC. Neurological function, as assessed by NDS, at 12, 24, 48 and 72 hrs after ROSC. Our results are consistent with those reported elsewhere^10, 11^.

Although some reports have shown similar neuroprotective effects of HS treatment during resuscitation, for example one randomised study of hypertonic saline infusion during resuscitation from out-of-hospital cardiac arrest in Germany^12^, very few studies have investigated the underlying mechanism for this phenomenon. In our study, for the first time, we have explored the mechanism of the neuroprotective effect and showed that hypertonic saline infusion suppressed apoptosis of hippocampal cells in a rat model of cardiopulmonary resuscitation.

The pathophysiologic mechanisms of ischemic brain damage caused by cardiac arrest and cardiopulmonary-cerebral resuscitation are complicated. These mechanisms consist of the primary insult of cardiac arrest (complete temporary global brain ischemia) and secondary derangements during and after reperfusion-reoxygenation^22^.

Sudden cardiac arrest can induce global brain ischemia and subsequently result in irreversible cerebral damage. Even after return of spontaneous circulation, reperfusion injury, cerebral edema and inflammation could exist for long time and lead to massive apoptosis of neuronal cells^17, 23^. It is well known that neuronal cell apoptosis is one of the main pathological change induced by CA post-ROSC, which is also the main form of brain cell death. Therefore, neuronal cell apoptosis may cause severe damage to neuronal function^5^. This impacts the post-resuscitation restoration of neuronal function. In our study, the apoptotic rate of CA-induced hippocampal neurons 72 hrs post-ROSC was up to 43%.

Hippocampal CA1 has been shown to be most vulnerable to ischemia in brain^24^ and especially, the neuronal cell apoptosis in Hippocampal CA1 after resuscitation is usually observed by H&E staining similar to our study. Suppression of apoptosis of hippocampal cells after cardiopulmonary resuscitation is an important strategy to ameliorate secondary neuronal dysfunction^7^. Results of Tunel assay and flow cytometry indicated anti-apoptotic effect of HS on Hippocampal CA1 neuronal cells after CA and CPR. Based on our study and previous works, the underlying mechanism maybe as follows:

1. HS treatment reduced cerebral edema and the damage of blood brain barrier.
  CA-induced brain ischemia could result in global cerebral edema, which may directly lead to high intracranial pressure during resuscitation. It is well known that lower intracranial pressure is associated with good prognosis post-ROSC^25^. Decompressive craniectomy was shown to decrease the apoptotic rate of neuronal cells in rat brain cells after CA and CPR^26^, also decreased the length of stay in the ICU in clinical^27^. Hypertonic saline, as a new dehydrating agent, has been applied in more and more studies^28^. A large body of evidence shows that hypertonic saline can significantly reduce intracranial pressure and enhance cerebral perfusion pressure^29^, whether in the early phase of post-ischemic global cerebral cell edema or in the later phase of vasogenic brain edema induced by impaired blood brain barrier^30^. HS was shown to be superior to mannitol, a traditional dehydrating agent, in attenuating cerebral edema, and working longer^31^.
  In addition, disruption of blood brain barrier was shown to play a major role in CA- induced brain injury by causing cerebral vascular insult^32^. The close association of cerebral edema and brain neuronal cell apoptosis with increased permeability of blood brain barrier is well-documented^33^. Importantly, previous hypertonic saline infusion was shown to ameliorate the disruption of blood brain barrier, protect integrity of cerebral vasculature and attenuate vasogenic brain edema^34^, which indicates HS to be a good agent for reducing intracranial pressure^35^, improving cerebral perfusion and for lowering neuronal apoptosis. Additionally, hypertonic saline was shown to inhibit the expression of vascular endothelial growth factor (VEGF), which is closely related to cerebral vascular integrity and is known to regulate the permeability of blood brain barrier. Besides, hypertonic saline has the protective effect on tight junctions within endothelial cells of brain vasculature^36^.
2. HS treatment improved cerebral blood perfusion.
  Hypotension after CA and CPR is known to exacerbate brain injury^37^. Maintenance of an optimal level of MAP (80–100 mmHg) is a key strategy for resuscitation. HS infusion induces immediate increase in blood osmotic pressure and effective blood volume and improves perfusion. Studies have showed that HS infusion maintains higher MAP by inducing sustained sympathetic nerve excitation, increases heart rate and augments strengthens myocardial contractility^10^. However, in our study, no significant difference was observed between the HS and HES groups with respect to post-resuscitation MAP. In contrast, a significant difference with respect to neuronal cell apoptotic rate was observed between the 2 groups; with the HES group showing a higher apoptotic rate (36.83%). Therefore, the mechanism of HS treatment which inhibited the neuronal cell apoptotic rate post-ROSC did not appear to be related to the MAP.
  Also, CA-induced global cerebral ischemia might have led to microvascular endothelial swelling and proliferation of the surrounding glial cells with swollen glial foot, which further induces compressive deformity and obstruction of the microvasculature. Many studies have shown that HS infusion regulates cerebral and cardiac microcirculation by attenuating cerebral cell edema, ameliorating microvascular endothelial swelling and reducing the microvascular resistance^9, 38^. We could speculate that the HS effect of increasing microcirculation may be one of the mechanisms of the anti-apoptotic effect of HS treatment on Hippocampal CA1 after CA and CPR.
3. HS treatment affected the expression of apoptosis protein.
  More and more attention is being paid on the relationship of apoptosis protein and cerebral injury^5^. Previous studies have shown significant changes in expressions of Bcl-2, Bax and caspase-3 proteins in cerebral neuron injury, while they exerted a significant effect on neuronal cell apoptosis. High expression of Bcl-2 had an anti-apoptotic effect. On the contrary, up-regulation of Bax increased apoptosis^39^. Some neuroprotective drugs can exert anti-apoptotic effects via regulating Bcl-2 and Bax expression during ischemia–reperfusion pathology process^40^. Bcl-2 and Bax form heterodimers, which play an important role in apoptosis, such as transduction of apoptotic signals. Caspase-3 is a key enzyme for activation of caspases of apoptosis pathways^41^. To some extent, changes in caspase-3 expression may reflect the degree of cell apoptosis^42^.
  In present study, Bcl-2 expression level in HS group was significantly higher than that in the NS and HES groups. Bax and Caspase-3 in the HS group were significantly lower than that in the NS and HES groups. Therefore, the anti-apoptotic effect of hypertonic saline infusion appears to have been mediated via up-regulation of anti-apoptotic protein and down-regulation of the pro-apoptotic proteins.
4. HS treatment ameliorated inflammation during resuscitation.

CA-induced cerebral ischemia triggers the host defense response to injury. Inflammation is one of the major pathological pathways involved in defense response to injury. Inflammatory cascades could be triggered within minutes of the onset of asphyxia^43^ and release inflammatory cytokines, such as tumor necrosis factor-α (TNF-α), interleukin-1 (IL-1) and monocyte chemotactic protein-1 (MCP-1)^13, 43^. Increasing evidences shows that HS can alleviate cerebral inflammation by inhibiting the release of microglia-derived inflammatory cytokines^13^. Besides, the protective effect of HS on the integrity of the blood brain barrier may also prevent inflammatory cytokines from entering the neurovasculature. It is possible that the attenuation of inflammation by HS treatment was associated with its’ anti-apoptotic effect. We did not evaluate the inflammation during resuscitation.

## Conclusion

In this study, HS treatment during resuscitation inhibited neuronal cell apoptosis in hippocampal CA1 at 72 hrs post-ROSC, and improved neuronal function as assessed by NDS. The likely mechanism of action is attenuation of cerebral edema; improvement in microcirculation perfusion; regulation of the balance of pro-apoptotic and anti-apoptotic proteins and alleviation of inflammation.

### Limitations

In our study, we studied the model of pretreatment with different fluids before cardiac arrest. But in clinical practice, cardiac arrest is more often due to cardiac etiology and always occurs unexpectedly^44^. In next study, extensive research will be performed using different cardiac arrest models from cardiac etiology and asphyxia using different fluid infusion. Also, we did not assess expressions of inflammatory cytokines in this study and the corresponding mechanism of the relationship of inflammation and apoptosis during resuscitation. Further research would be needed to evaluate these aspects.

## Electronic supplementary material

Supplementary Information
