# Supplementary material for: Hypertonic saline infusion suppresses apoptosis of hippocampal cells in a rat model of cardiopulmonary resuscitation
Source: Sci Rep. 2017 Jul 19;7:5783. doi: 10.1038/s41598-017-05919-4 (PMC5517425; doi:10.1038/s41598-017-05919-4)
Supplement: Supplementary file 1 — Supplementary Information [file 41598_2017_5919_MOESM1_ESM.pdf]

Hypertonic saline infusion suppresses apoptosis of hippocampal cells in a rat model of cardiopulmonary resuscitation

Xiang Zhou [1,2], Yong Liu [2], Yang Huang [1], ShuiBo Zhu [1,2], Jian Zhu[2], RongPing Wang[2]

1 Southern Medical University, Guangzhou, China; 2 Department of Thoracic Cardiovascular Surgery, Wuhan General Hospital of People's Liberation Army of China

Corresponding author: ShuiBo Zhu, whzyyzsb@126.com. Postal address : wuluo road, 627#, Wuhan, Hubei, China, 430070.

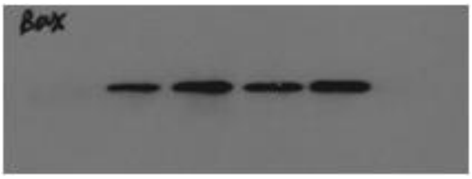
Bax

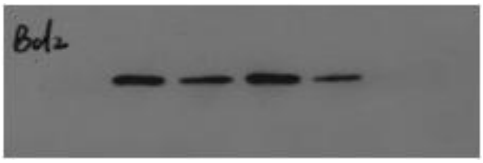
Bcl2

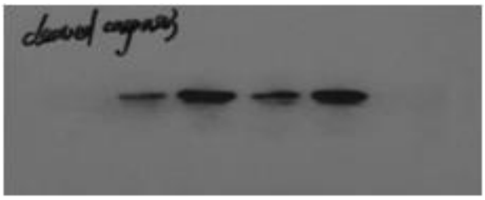
cleaved caspase3

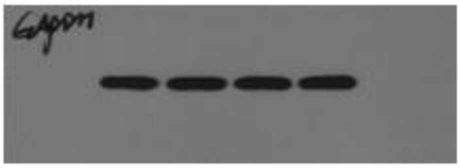
GAPDH
